# Urogenital anomalies in children with anorectal malformations: a single institution observational study

**DOI:** 10.3389/fsurg.2025.1497644

**Published:** 2025-04-24

**Authors:** Belachew Dejene Wondemagegnehu, Solomon Wubetu Asfaw

**Affiliations:** ^1^ College of Health Sciences, Addis Ababa University, Addis Ababa, Ethiopia; ^2^ College of Medicine and Health Science, Jijiga University, Jijiga, Ethiopia

**Keywords:** anorectal malformation, urinary, genital, urogenital anomalies, renal anomalies

## Abstract

**Introduction:**

Anorectal malformations (ARM) consist of a range of anomalies that occur in approximately 3.5 in 10,000 live births. Though variable, about half of these patients present with an associated genitourinary abnormality. Considering this high prevalence, this study aimed to assess the specific occurrence of urogenital anomalies in patients with anorectal malformations.

**Methods:**

An institution-based observational study was conducted on 156 patients with anorectal malformation, all of whom were screened for urogenital anomalies. Data were collected using a pre-structured questionnaire and analyzed using SPSS (IBM) Version 26 software. Relevant statistical analysis was performed, and the results are presented in tables.

**Results and discussion:**

Of the 156 patients with ARM studied for associated urogenital anomalies, 91 (58.3%) were females with a male-to-female ratio of 0.7:1 and a median age of 12 months (IQR = 1–24). Forty-six of them (29.5%) had urogenital anomalies, of whom 22 (14.1%) had isolated urologic anomalies and 20 (12.8%) had both urologic and genital anomalies. Renal anomalies were found in 34 (21.8%) patients. The association between gender and genital anomalies was significant, *χ*^2^ (1), *N* = 156 = 4.09, *p* = 0.04. The type of ARM has a highly significant association with genital anomalies *χ*^2^ (11), *N* = 156 = 21.95, *p* = 0.009. Males were less likely to exhibit urogenital anomalies [OR = 0.386, 95% CI (0.15–0.995), *p* = 0.048]. Children with complex ARM have 3.4 times genital and 2.3 times urinary anomalies than less complex forms. In summary, urogenital anomalies are the most common anomalies occurring in association with anorectal malformation. Genital anomalies have an association with gender with more occurrence in females. Children with complex anorectal malformations have a higher chance of urogenital anomalies.

## 1 Introduction

Anorectal malformations (ARM) consist of a range of anomalies that occur in approximately 3.5/10,000 live births (1, 2). Defects range from very minor and easily treatable to complex and difficult to manage. These defects are often associated with other anomalies (3). The incidence rate of these anomalies differs by the type of ARM, ranging from 20% to 80% across different literatures, and involves the genitourinary, cardiac, musculoskeletal (vertebral, spinal, limb), and gastrointestinal systems (4). Genital and urinary (GU) anomalies occur with an overall incidence rate of 30%–80% and are higher in children with complex ARM (5, 6).

The presence of associated congenital anomalies can lead to increased overall mortality and serious morbidity (5, 7). Children with these anomalies often experience a poorer quality of life as compared with children without associated anomalies which influence the prognosis for sexual function and fertility (7, 8).

Routine screening of newborns should be performed to evaluate associated defects, which include esophageal atresia, cardiac abnormalities, and renal anomalies (4). Urogenital anomalies are the most commonly associated anomalies in patients with ARM. In the studies of van der Steeg et al. and Sabzehei et al., a significant number of cases screened for urinary anomalies exhibited high-grade vesicoureteral reflux (VUR). Based on this finding, the authors recommended routine VUR screening in all patients with anorectal malformation (9). Timely assessment is necessary to identify anomalies requiring intervention and to prevent complications such as urinary tract infection (UTI), renal scarring, and subsequent renal failure (9).

Although urinary and genital anomalies are the most commonly associated anomalies found during the general evaluation of patients with anorectal malformations, there is limited literature on the rates of screening and identification specific to urogenital anomalies. This observational study aimed at assessing the prevalence rate of occurrence of associated urogenital anomalies in patients with anorectal malformations based on clinical, operative, and imaging findings.

## 2 Materials and methods

### 2.1 Study setting and period

The study was conducted from February 2018 to January 2020 in Tikur Anbessa Tertiary Teaching Hospital, Addis Ababa, Ethiopia, where a significant number of pediatric colorectal and urologic cases are managed by residents, fellows, and consultants.

### 2.2 Source population

All children admitted for the management of Anorectal malformation during the study period.

### 2.3 Study participants and selection

A total of 162 children were admitted for the management of anorectal malformations during the study period. Regardless of their antenatal or postnatal conditions, 156 of these patients were included in the study and screened for the presence of associated genital and urinary anomalies, and the remaining 6 patients were excluded because of incomplete documentation. All selected cases underwent an ultrasound examination. However, due to financial constraints, voiding urethrocystography (VUCG) and intravenous pyelography (IVP) were only performed on those with ultrasound findings requiring further investigation. In this context, VUCG was performed for individuals suspected of having VUR with hydroureteronephrosis on ultrasound, while IVP was utilized to exclude the possibility of pelviureteric junction obstruction (PUJO) in individuals where no demonstrable proximal ureter but moderate to severe hydronephrosis is identified. Magnetic resonance urography (MRU), renal scans, and urodynamics were not performed in this study, as they were not affordable for our patients. Lesions were classified using the Krickenbeck classification (10).

### 2.4 Study design

The study was an institution-based observational study.

### 2.5 Study variables

Age, gestational age, sex, birth weight, type of anorectal malformation, type of urinary and genital anomalies, and type of screening done.

### 2.6 Data quality control measures

The investigators thoroughly examined patient charts and reviewed clinical findings to gather as much information as possible. The questionnaires were filled out by the principal investigator during ward rounds.

### 2.7 Data collection tool

A structured set of questionnaires was designed according to the preset objectives. Data regarding patient demographics, the type of ARM, and associated anomalies were collected via clinical examination, imaging, and reviewing operation findings. For this study and to facilitate comparison between studies in the literature, abnormalities were categorized as genital and urinary. An ultrasound was performed to rule out urinary and genital anomalies. Voiding cystourethrogram was conducted for patients with identified genitourinary abnormalities during ultrasound investigation. Other relevant investigations were also carried out to look for associated anomalies.

### 2.8 Data processing, analysis, and interpretation

For this study and to facilitate comparison between studies in the literature, abnormalities were categorized as genital and urinary. Data were analyzed using SPSS (IBM) for Windows version 26 statistical software. The chi-square test and logistic regression were used to analyze associations and relations among the study variables. The results are presented in tables and charts. A *p*-value of <0.05 was considered statistically significant where applicable.

### 2.9 Ethical statement

Ethical clearance was obtained from the research ethics committee of the Department of Surgery and the Institutional Review Board (IRB) of the College of Health Sciences, Addis Ababa University, with Ref. No 161/19/11. Since the study was conducted based on routine care of patients, the need for consent to participate was waived by the same committee. However, verbal informed consent was obtained from parents after the objective of the study was explained to them. Confidentiality was maintained throughout the research undertaking.

## 3 Results

A total of 156 patients of ARM were studied for associated urogenital anomalies, of whom 91 (58.3%) were females with a male-to-female ratio of 0.7:1 and a median age of 12 months (IQR = 1–24). The majority, 92/156 (58.9%), presented during the first year of life. Forty-one patients (26.3%) presented during neonatal age, and 147/156 (94·2%) of them were term infants. Normal birth weight was recorded in 91%, and 49 (31.4%) were firstborn (Table 1).

**Table 1:** Demographic characteristics of children with anorectal malformation screened for urogenital anomalies.

Variables	Characteristics	Frequency	Percent
Sex	Male	65	41.7
	Female	91	58.3
Age	Neonatal	41	26.3
	Infants	51	32.7
	Children 1–4 year	64	41
Birth weight (gms)	<1,500	1	0.6
	1,500–2,500	13	8.3
	>2,500	142	91
Parity	Para-I	49	31.4
	Para-II	48	30.8
	Para-III and above	59	37.8

Regarding their presentation, all patients presented with features of ARM, and 74 (47.4%) had a VACTERL association. During screening by physical examination, the most common urogenital findings in boys were hypospadias 7/65 (10.8%), with four of these being proximal and associated with the bifid scrotum, and undescended testis 7/65 (10.8%). In girls, the most common findings were vaginal septum in 7/91 (7.7%) and hydrocolpus. Among the patients, 46.1% had complex malformations with the majority 61.1% being male (*p* < 0.001) (Table 2).

**Table 2:** Severity of anorectal malformations distribution with gender.

Gender	Complex	Less complex	Total
Male	44 (28.2%)	21 (13.4%)	65 (41.7%)
Female	28 (17.9%)	63 (40.4%)	91 (58.3%)
Total	72 (46.1%)	84 (53.8%)	156 (100%)

Forty-six patients (29.5%) had urogenital anomalies, of whom 22 (14.1%) had isolated urologic anomalies, 20 (12.8%) had both urologic and genital anomalies, and only 4 had isolated genital anomalies. Among the urinary anomalies, renal anomalies were the most common, found in 34 (21.8%) of patients. Hydronephrosis and renal agenesis were the most common renal findings (Table 3).

**Table 3:** Type of urologic anomalies found during screening in 156 children with anorectal malformation.

Type of anomaly	Frequency	Percentage
Crossed fused ectopia	2	1.3
Dysplastic kidney	1	0.6
Ectopic kidney	2	1.3
Ectopic kidney with hydronephrosis	1	0.6
Horseshoe kidney	2	1.3
Hydronephrosis alone	11	7.1
Hydronephrosis with vesicoureteral reflux	1	0.6
Hydronephrosis with Megaureter	1	0.6
Hydronephrosis with ureterocele	1	0.6
Malrotated renal pelvis	1	0.6
Medullary sponge kidney	1	0.6
Renal agenesis	10	6.4
Total with renal anomalies	34	21.8

Voiding cystourethrography was performed on nine patients, six of whom underwent further workup for hydronephrosis, one with unremarkable results and five with vesicoureteral reflux which accounts for 14.7% of those with renal anomalies. Intravenous pyelography (IVP) was performed on five patients, two of whom had megaureter, two had non-excreting kidneys, and one had a duplex system with ureterocele.

The sacral ratio (SR) was assessed, and 57.5% of the patients showed SR > 7, and 48.5% of those with urinary anomalies and 50% of those with genital anomalies had SR ≤ 7 (Table 4).

**Table 4:** Mean sacral ratio in relation to genitourinary anomalies and type of anorectal malformations.

Type of ARM (Krickenbeck classification)	Genitourinary anomalies		Total		Mean sacral ratio
	Yes	No	*n*	%	
Perineal fistula	6	21	27	17.3	0.73 ±0.12
Recto-urethral (bulbar)	10	15	25	16.0	0.73 ±0.08
Recto-prostatic/bladder neck	2	4	6	3.8	0.58 ±0.19
ARM without fistula	4	12	16	10.3	0.73 ±0.0.1
vestibular fistula	11	46	57	36.5	0.71 ±0.12
Vaginal fistula	1	2	3	1.9	0.76 ±0.01
Persistent cloaca	7	9	16	10.3	0.67 ±0.22
Rectal atresia/stenosis	1	1	2	1.3	0.73 ±0.1
Pouch colon	3	0	3	1.9	0.72 ±0.1
Others	1	0	1	0.6	0.35
Total	46	110	156	100	

A chi-square test of independence was performed to evaluate the association between gender and incidence of urinary and genital anomalies in children with ARM. The relationship between gender and the occurrence of genital anomalies was significant, *χ*^2^ (1), *N* = 156 = 4.09, *ρ* = 0.04. However, there was no association between gender and the presence of urinary anomalies. In the same way, the type of ARM was found to have a highly significant association with the occurrence of genital anomalies, *χ*^2^ (11), *N* = 156 = 21.95, *p* = 0.009.

A logistic regression was performed to look for a relationship between the type of ARM and the presence of urogenital anomalies. Children with complex ARM were more likely to have associated urogenital malformation than those with less complex malformations. Males were less likely to exhibit urogenital anomalies than females [OR = 0.386, 95% CI (0.15–0.995), *p* = 0.048]. Children with complex anomalies have 3.4 times genital and 2.3 times urinary anomalies than those with less complex ARM [OR = 3.421, 95% CI (1.250–9.360), *p* = 0.01, and OR = 2.333, 95% CI (1.154–4.719), *p* = 0.01, respectively].

## 4 Discussion

The research indicated a marginal female majority, with approximately 50% presenting associated urogenital anomalies. A notable correlation was found between the presence of urogenital anomalies and complex anorectal malformations, with renal anomalies being more prevalent among those with urological conditions.

In this cohort, the proportion of male to female patients was slightly higher, with a ratio of 0.7:1. This aligns with findings from studies conducted by Fuchs et al., Mfinanga et al., and Gama and Tadesse (6, 11, 12), yet contrasts with studies by Ford et al., Oh et al., Sabzehei et al., and Chanchlani et al., which observed a male predominance (1, 5, 9, 13). The literature does not provide clear explanations for these gender discrepancies, highlighting the need for further research.

The median age at the time of initial diagnosis was 12 months. The majority of them (58.9%) were diagnosed within the first year of life, including 26.3% during the neonatal period. This rate is higher than that reported in similar studies. Additionally, a significant majority (94.2%) were full-term infants, with 91% recorded as having a normal birth weight (9, 11). These findings suggest specific patterns in the presentation and characteristics of the condition that merit further investigation.

In this study, all patients exhibited anorectal malformation symptoms with 47.4% also presenting with VACTERL association, aligning with certain literature ranging from 9% to 44% (14, 15). The most frequent urogenital abnormalities observed in males were hypospadias and cryptorchidism, each at 10.8%, while in females, the vaginal septum was noted in 7.7% of cases, a figure lower than reported in similar studies but comparable to the study by Forlini et al. (4, 16, 17).

Complex malformations were identified in 46.1% of patients, predominantly male (61.1%), exceeding the 22.7% incidence reported by Moras et al. (18) Renal anomalies were detected by ultrasound in 34 (21.8%) of which hydronephrosis (15/34; 44.1%), renal agenesis (10; 29.4%), and vesicoureteral reflux (VUR; 6%) are the most common findings, consistent with another research (16, 19).

Urologic anomalies are recognized as one of the most frequent associations with ARM. Our baseline assessment for genitourinary anomalies included renal and pelvic ultrasound, which was performed on all patients. The findings revealed that 29.5% had urogenital anomalies, with 16% having urologic and 13.5% genital anomalies, which are higher than the 18.75% reported by Ali et al., but fall within the 10.3%–40% range reported in Bjoersum-Meyer et al.'s systematic review, and are less than the 30% urinary anomalies reported by Duci et al. (16, 20, 21). The lower incidence in our study may be due to the significant number of patients not routinely screened for vesicoureteral reflux as recommended by Sabrina et al. (22). The most prevalent urologic anomalies identified were hydronephrosis in 9.5% and renal agenesis in 5.7% of 156 ARM patients.

In this study, patients with perineal fistula exhibited a better sacral ratio compared with those with recto-prostatic/bladder neck fistula, aligning with the findings reported by Chen et al. (23). Additionally, recto-bulbar fistula demonstrated a better sacral ratio than the more proximal anomalies.

A significant correlation was found between gender and the presence of genital anomalies, yet no such correlation was observed for urinary anomalies. The severity of ARM showed a strong link with the occurrence of genital anomalies, with complex anomalies being highly associated, ranging from 30% to 52%, with the presence of urogenital abnormalities (6, 13, 16, 19, 20, 24).

In our study, patients with anorectal malformations commonly presented with urogenital anomalies. Children with complex anorectal malformations exhibited 3.4 times increased occurrence of urogenital anomalies. However, the incidence of renal anomalies was comparatively lower, indicating the necessity for a thorough diagnostic approach, including voiding urethrocystography, especially for patients with complex anorectal malformations. For more complex ARMs, based on availability, a systematic approach to screening should include initial routine renal and bladder ultrasound to identify gross anomalies, VCUG for detecting reflux or bladder outlet obstruction, and MRU for detailed anatomical assessment.

The study assessed only the incidence of ARM-associated urogenital anomalies and their relationship with the complexity of ARM. Further management and their outcome results will follow in the future. Limitations encountered were problems in some patients' documentation of follow-up and retrieval of some results from the database. Additionally, magnetic resonance urography, renal scans, and urodynamics were not performed, as they were not affordable for our patients. These would have shown the true multiple anatomic and functional abnormalities in the patients (25).

The findings in this research have identified not only the commonest associated urogenital anomalies but also supposed to be rare genital anomalies, such as proximal hypospadias with bifid scrotum, in noticeable numbers. This underlines the significance of screening patients with anorectal malformation to improve the detection rate and overall outcomes of these children with associated urogenital anomalies.

## Data Availability

The raw data supporting the conclusions of this article will be made available by the authors, without undue reservation.
