# Supplementary material for: Urogenital anomalies in children with anorectal malformations: a single institution observational study
Source: Front Surg. 2025 Apr 24;12:1497644. doi: 10.3389/fsurg.2025.1497644 (PMC12058842; doi:10.3389/fsurg.2025.1497644)
Supplement: Supplementary file 1 [file Datasheet1.pdf]

STRUCTURED QUESTIONER FORMAT

Patient Medical Registration No.: ……………………………….Phone no**…………………………….**

No	Variables	Answers		
	**1. Patient Demographics**			
1.1	*Age at initial presentation*	☐ Birth -3 days	☐ 4 days -30 days	☐ 31days- 12 months
		☐ 1 year - 5 years	☐ 5 years -14 years	
1.2	*Gender*	☐ Male	☐ Female	
1.3	*Gestational Age*	☐ < 37 weeks	☐ > 37weeks	
1.4	*Birth Weight*	☐ <1500 gram	☐ 1500-2500 gram	☐ >2500 gram
1.5	*Address*	☐ Addis Abeba ☐ Oromia<br>☐ Harar ☐ Somali	☐ Amhara<br>☐ Tigray	☐ SNNP<br>☐ Other region

2. Mode of Presentation

- ☐ Features of intestinal obstruction without any sign of fistula
- ☐ Passage of meconium through abnormal routes without significant obstruction
- ☐ Passage of fecal matter through abnormal routes with obstruction
- ☐ Symptoms of other associated anomalies as compliant

3. Screening by Physical examination

3.1.. HEENT/RESP	☐ Any Dysmorphic feature seen,<br>☐ Excessive oral secretions/choking during feeding,<br>☐ Failure to pass a Nasogastric/Orogastric tube
3.2. CVS	☐ Murmur ☐ Cyanosis ☐ Diaphoresis with feeds ☐ Respiratory distress
3.3. GI	☐ Abdominal wall defect
3.4. GUS	☐ Abdominal mass (hydrocolpos) ☐ Hypospadias<br>☐ Undescended testes ☐ bifid scrotum ☐ Vaginal septum
3.5. MSK/SPINE	☐ Abnormal Spine curvature /shape , Hairy patch<br>☐ Sacral dimple , Abnormal neurologic exam<br>☐ Radial & thumb anomalies ,Polydactyly

4. Screening by Investigations

4.1.	Cardiac/ Respiratory	☐ Echocardiography (if indicated)<br>☐ Doppler u/s (if indicated)<br>☐ CXR (if indicated)	☐ Unremarkable<br>☐ Anomaly, specify<br>…………………………
4.2.	Genitourinary	☐ Renal, Pelvic U/S (all)<br>☐ VCUG (if indicated)<br>☐ MRU (if indicated)	☐ Unremarkable<br>☐ Anomaly, specify<br>……………………..
4.3.	Musculoskeletal	☐ Lumbosacral spine and Pelvic x-ray(all)<br>☐ Spinal US (< age 3months) (all)<br>☐ MRI (> age 3mn) if indicated /afford<br>☐ Limb x-ray (if indicated)	☐ 1.Unremarkable<br>☐ Anomaly, specify<br>……………………..
4.4	Other GI	☐ Abdominal u/s (all)<br>☐ Plain abdominal x ray (if indicated)<br>☐ Contrast studies (if indicated)<br>☐ Distal colostrogram (if they can afford)	☐ Unremarkable<br>☐ Anomaly, specify<br>……………………..

5. Type of ARM

5.1.	Type of ARM<br>(Krickenbeck's class)	☐ Perineal fistula<br>☐ Rectourethral (Bulbar) fistula<br>☐ Rectourethral (Prostatic) fistula<br>☐ Recto bladder neck fistula<br>☐ Vestibular fistula<br>☐ Persistent cloaca<br>☐ ARM without fistula<br>☐ Rectal atresia<br>☐ Complex defects<br>☐ Others …………………………….
5.2.	Type of associated anomaly/syndrome identified	☐ Cardiac ………………………………………………………..<br>☐ Genito-urinary ……………………………………………………..<br>☐ Musculoskeletal /spine……………………………………………..<br>☐ Other GI……………………………………………………………<br>☐ Syndromes……………………………………………………….
